# Optimization criteria for ordering myeloid neoplasm next‐generation sequencing

**DOI:** 10.1002/jha2.1036

**Published:** 2024-10-23

**Authors:** Savanah D. Gisriel, John G. Howe, Christopher A. Tormey, Richard Torres, Karl M. Hager, Henry M. Rinder, Alexa J. Siddon

**Affiliations:** ^1^ Department of Pathology Yale School of Medicine New Haven Connecticut USA; ^2^ Department of Laboratory Medicine Yale School of Medicine New Haven Connecticut USA; ^3^ Department of Internal Medicine Section of Hematology Yale School of Medicine New Haven Connecticut USA

**Keywords:** MDS, myeloid neoplasms, next‐generation sequencing

## Abstract

**Introduction:**

Myeloid neoplasms (MNs) frequently harbor pathogenic mutations not detected by karyotyping and fluorescence in situ hybridization; hence, next‐generation sequencing (NGS) is necessary for diagnosis, risk stratification, and therapy. If, however, NGS is not clinically indicated but still performed, the results may promote futile avenues of investigation, heighten patient distress, and increase cost.

**Methods:**

We created criteria to approve NGS testing for MN (MN‐NGS) with the goal of maximizing actionable results. These actionable results include making a new MN diagnosis, characterizing a MN with baseline mutational status, and altering treatment plans. Approval criteria included clinical suspicion of new, relapsed, or worsening disease and end‐of‐induction chemotherapy. Cancellation criteria included the suspicion of non‐myeloid disease only, no suspicion of progression of a known MN, no evidence for recurrence post‐transplant, a diagnosis of chronic myeloid leukemia, and cases using blood when a concurrent bone marrow NGS is being performed. We applied these criteria to NGS tests ordered at our institution between August and December 2018 and determined whether any tests meeting our cancelation criteria yielded actionable results.

**Results:**

Consecutive MN‐NGS orders (*n* = 174) were retrospectively categorized as appropriate (Group A, *n* = 115), inappropriate (Group B, *n* = 29), and appropriately canceled (group C, *n* = 30). Seventy‐five of the 115 (65%) Group A tests and none of the 29 (0%) Group B tests yielded actionable results (*p* < 0.0001).

**Conclusion:**

Approximately one third (59/174) of MN‐NGS test orders can be safely canceled using these criteria, which would result in $150,370 of Centers for Medicare and Medicaid Services‐reimbursed savings annually.

## 1 INTRODUCTION

Myeloid neoplasms (MNs), including acute leukemia, myelodysplastic syndromes/neoplasms, and myeloproliferative neoplasms, are increasingly classified according to their mutational profile since certain molecular signatures are now known to carry diagnostic, therapeutic, and/or prognostic significance [1]. Next‐generation sequencing (NGS) for MNs can identify mutations not routinely detected by conventional karyotype or fluorescence in situ hybridization [2]. Hence, NGS data for MNs provide high‐quality stratification for risk and/or therapy including identifying clonality in triple‐negative myeloproliferative neoplasms (MPNs) [3, 4], predicting overall survival in myelodysplasia (MDS) patients [5], and monitoring for minimal/measurable residual disease (MRD) following induction [6, 7]. As a result, NGS for MNs is now an integral component of clinical practice.

The interpretation and implementation of NGS results are rapidly evolving, particularly as we gain insight into the importance of molecularly defined MNs [8, 9]. Challenges include discriminating leukemia‐related mutations from polymorphisms or passenger mutations, classifying somatic variants as leukemia‐related versus clonal hematopoiesis of indeterminant potential‐related, identifying pathogenic germline alterations, distinguishing true variants from sequencing artifacts, and assessing for MRD given variant allele frequency thresholds that may limit sensitivity [9]. When NGS is performed on a patient in which there is no clear clinical suspicion or laboratory‐based indication of risk for a new, progressive, or relapsed MN, integration of findings can potentially lead to futile avenues of investigation and a potentially inaccurate view of the patient's risk. Further, inappropriately ordered tests compromise the efficient allocation of time and resources and may unnecessarily increase patient distress. To the authors’ knowledge, this is the first study that (1) applies objective criteria to approving NGS testing for acute leukemia, myelodysplastic syndromes/neoplasms, and myeloproliferative neoplasms (MN‐NGS), (2) quantifies the degree of inappropriate ordering, and (3) explores the financial implications of testing when approval criteria are not met.

## 2 MATERIALS AND METHODS

### 2.1 Criteria for NGS approval and cancelation

Using clinical experience and evidence‐based indications for MN evaluation, approval and cancelation criteria for MN‐NGS orders were constructed (Table 1) and retrospectively applied to each test order. Given its myeloid origin, blastic plasmacytoid dendritic cell neoplasm was also considered to be an acceptable indication for ordering the MN‐NGS panel (Table 1) [10, 11]. Orders were considered appropriate if they met at least one approval criterion and no cancelation criteria, and orders were considered inappropriate if they met at least one cancelation criterion.

**TABLE 1:** Criteria to perform or cancel next‐generation sequencing (NGS) panel.

NGS criteria	#	Description
Criteria to perform	1	New diagnosis or clinical suspicion of AML (marrow preferred, blood acceptable if circulating blasts^a^) [12]
	2	New diagnosis or clinical suspicion of MDS with increased blasts or blastic plasmacytoid dendritic cell neoplasm (BPDCN;^c^ blood or marrow)^b^
	3	New diagnosis or clinical suspicion of MDS without increased blasts after otherwise negative workup (marrow preferred^a^)^b^
	4	Clinical‐ or laboratory‐based concern for relapse or progression of AML/MDS/MPN/other myeloid neoplasm (MN)^c^ after a period of normal or stable findings by flow cytometry/surgical pathology (blood or marrow)
	5	New diagnosis or clinical suspicion of *BCR::ABL1*‐negative MPN (blood or marrow)^d^
	6	End of induction chemotherapy for new AML or high‐risk MDS (no less than 30 days and no more than 60 days post start of chemotherapy and no known measurable disease by flow cytometry or surgical pathology, marrow preferred^a^) [12]
Criteria to cancel	7	Diagnosis is a non‐myeloid disease, or there is no suspicion for AML/MDS/MPN/other MN^b^
	8	Patient had NGS performed within 6 months, there is no significant change in disease by flow cytometry or surgical pathology, and there is no clinical suspicion of progression
	9	Patient had a stem cell transplant, last donor chimerism is >95% donor, and there is no evidence for recurrence on flow cytometry or surgical pathology
	10	Diagnosis of CML with no concern for AML
	11	No above cancelation criteria met, but no approval criteria met
	12	Sample type is blood, but test is already being performed on concurrent marrow

Abbreviations: AML, acute myeloid leukemia; CML, chronic myeloid leukemia; MDS, myelodysplasia; MPN, myeloproliferative neoplasm; MRD, minimal/measurable residual disease.

^a^ Blood acceptable only when patient‐centered reasons preclude marrow assessment.

^b^ Performing NGS is still acceptable if there is already an established diagnosis of MN, but the panel has never been previously performed.

^c^ While BPDCN is not considered a MN, the mutational landscape of BPDCN is similar to that of MNs and BPDCN has been found to be associated with MNs [10]. Thus, BPDCN is considered an appropriate diagnosis for which to perform the AML/MDS NGS panel.

^d^ If CML is also in the differential but *BCR*::*ABL1* results are pending, it is suggested to wait until the *BCR*::*ABL1* results are finalized before proceeding with NGS. If *BCR*::*ABL1* is positive, cancelation criterion #10 will be employed.

### 2.2 Definition of actionable results

MN‐NGS orders were considered actionable if, as a result of being performed: (a) a new MN diagnosis was able to be made, (b) a newly established or newly recurrent MN diagnosis was characterized with baseline mutational status for follow‐up purposes, or (c) the treatment plan changed.

### 2.3 Data collection and patient cohort

Consecutive MN‐NGS orders for all adult patients (≥16 years of age) received from August 14 to December 14, 2018, were retrospectively reviewed. NGS orders were categorized according to our criteria as appropriate (Group A), inappropriate (Group B), and appropriately canceled (Group C). The diagnoses and variant characteristics of Groups A and B were compared. The following information for each NGS order was reviewed: sample type (blood or bone marrow [12]), clinical history, order indication, cytomorphologic and flow cytometric diagnosis if available, NGS findings if the test was performed, and clinical follow‐up. Tests that were canceled by the ordering provider before testing despite meeting our criteria to be approved were excluded.

### 2.4 NGS

NGS was performed on blood or bone marrow samples. Genomic DNA was extracted from patient samples and amplified by multiplex polymerase chain reaction (PCR) using a custom set of primers that was developed using Ion Torrent Ampliseq software (Thermo Fisher Scientific). The primer set targets specific hotspot regions, as well as entire exons, of 49 genes included in a custom gene panel designed for myeloid neoplasia. The DNA was amplified using 1298 primer pairs covering 99.6% of the exonic regions of the genes. Ampliseq libraries were prepared and quantitated by real‐time PCR.

NGS was performed on the Ion Torrent S5 Sequencer (Thermo Fisher Scientific) and analyzed with the Torrent Suite Software. Variant calling was performed using Ion Reporter Software. Variant classification was generally based on the American College of Medical Genetics and Genomics Sequence variant interpretation guidelines [13]. Pathogenic variants were annotated using multiple modalities including COSMIC (https://cancer.sanger.ac.uk/cosmic) and ClinVar (https://www.ncbi.nlm.nih.gov/clinvar/), among others. Variants were considered to be Level 1 based on widely accepted diagnostic, prognostic, or therapeutic significance, while Level 2 pathogenic variants had less known clinical significance but based on the type or position of the variants were considered to be related to disease or if they were reported in a smaller number of MNs. The variant nomenclature followed conventions recommended by the Human Genome Variation Society (http://www.hgvs.org/mutnomen/).

### 2.5 Single mutation analysis

Separate non‐NGS *JAK2* V617F and *FLT3* mutation analyses were performed in select cases on the identical NGS sample. *JAK2* V617F mutation analysis was performed using multiplex PCR followed by gel electrophoresis, while *FLT3* ITD and D835 mutation analysis was performed using multiplex PCR followed by capillary electrophoresis.

### 2.6 Statistical analysis

Statistical analysis was performed using GraphPad Prism 6 software (GraphPad). Two‐tailed *p*‐values were calculated using Fisher's exact test. A *p*‐value of less than 0.05 was considered statistically significant.

## 3 RESULTS

**FIGURE 1:**
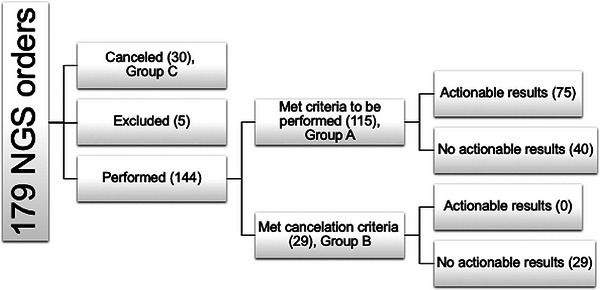
Grouping of next‐generation sequencing (NGS) orders. AML, acute myeloid leukemia; MDS, myelodysplasia.

**FIGURE 2:**
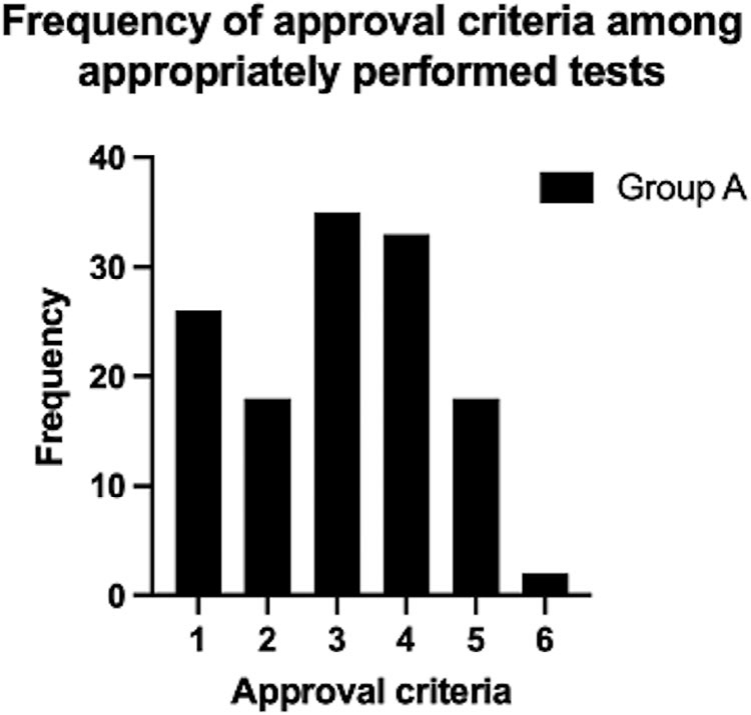
The frequency of each approval criterion among Group A tests. Criteria are defined in Table 1.

**FIGURE 3:**
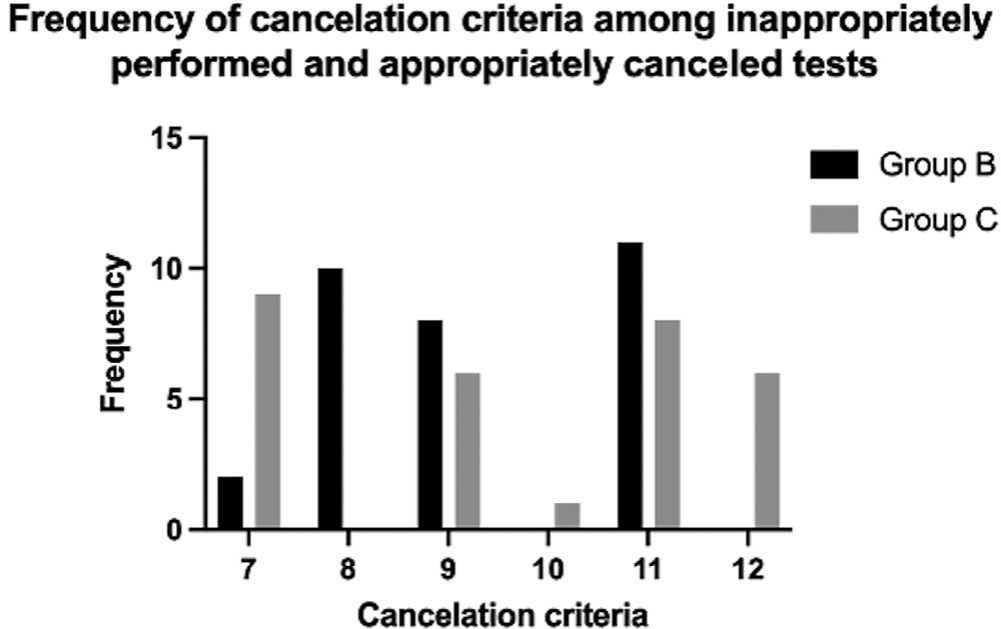
The frequency of each cancelation criterion among Group B and Group C tests. Criteria are defined in Table 1.

Of the 179 consecutive MN‐NGS orders, five were excluded because of up‐front clinician cancellation. Thirty orders were appropriately cancelled in the laboratory using our criteria (Group C; Figure 1). Of the 144 tests performed, 115 met the approval criteria (Group A), while 29 met the cancellation criteria (Group B; Figure 1). The approval criterion most applicable to Group A was criterion #3, that is, there was a new diagnosis or clinical suspicion of MDS without increased blasts after an otherwise negative workup (Table 1; Figure 2). The cancellation criterion most frequently applied to Group B was #11, that is, the order did not meet any approval criteria (Table 1; Figure 3), while cancellVation criterion #7 was most frequently applied to Group C, that is, the diagnosis was a non‐myeloid disease or there was no suspicion for an MN (Table 1; Figure 3).

**FIGURE 4:**
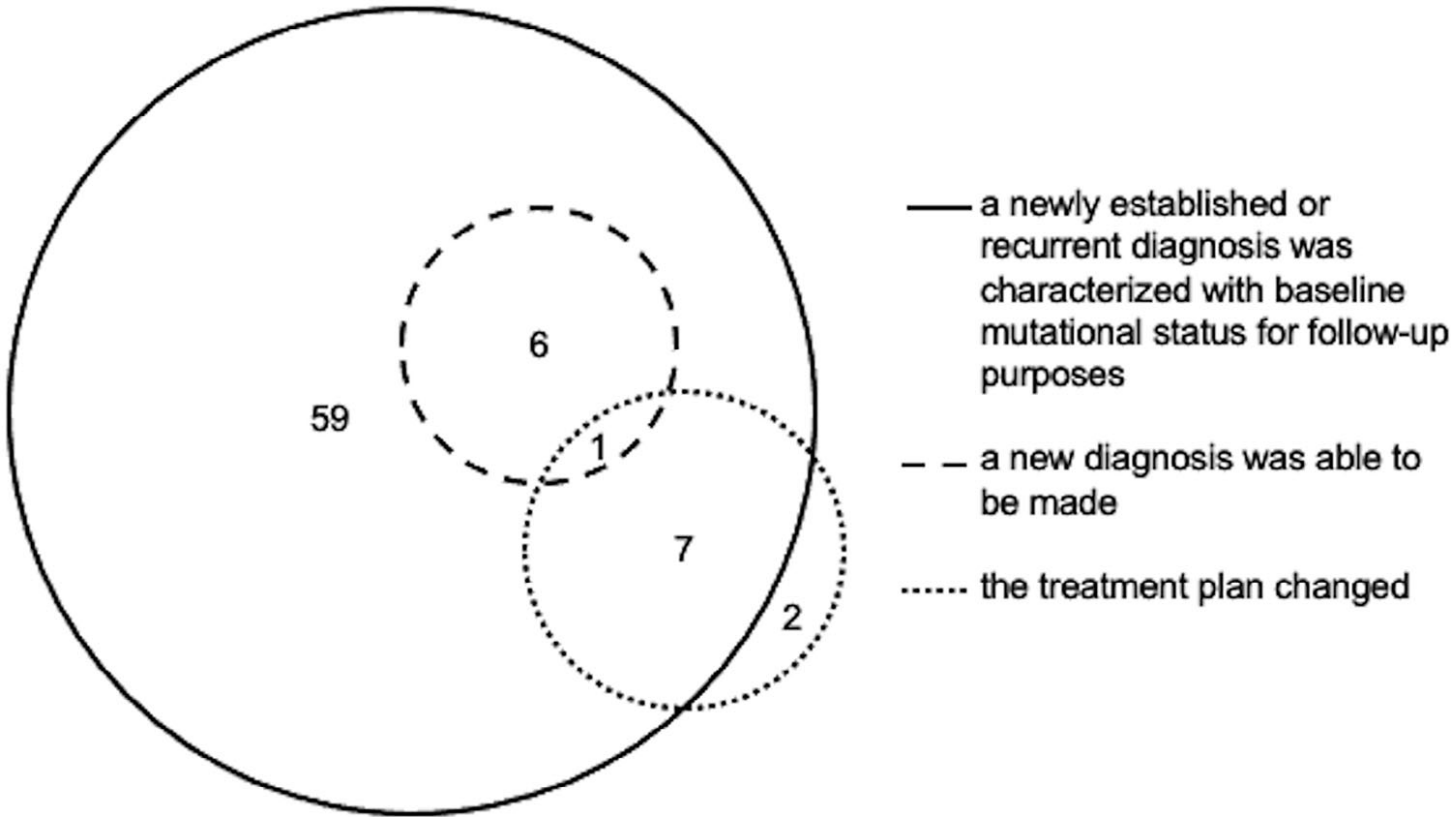
Venn diagram illustrating the distribution of different actions that were taken as a result of each actionable NGS test.

Seventy‐five of the 115 (65%) appropriate Group A tests and none of the 29 (0%) inappropriate Group B tests yielded actionable results (*p* < 0.0001; Table 2). Among the 75 actionable results, the most common outcome was that a new or newly recurrent diagnosis was characterized with baseline mutational status for follow‐up purposes (73/75, 97%; Figure 4). Other actionable outcomes included the establishment of a new diagnosis (7/75, 9.3%) and change in treatment (10/75, 13%; Figure 4).

**TABLE 2:** Patient and test characteristics among appropriately and inappropriately ordered AML/MDS NGS tests.

Patient and test characteristics	Appropriately ordered (Group A: 115 tests)	Inappropriately ordered (Group B: 29 tests)	*p‐*value
Median age, years (range)	70 (24–92)	69 (42–90)	
Sex (male/female)	69/46	18/11	1.000
Test to evaluate for AML	50/115 (43.5%)	14/29 (48.3%)	0.680
Test to evaluate for MDS	60/115 (52.2%)	11/29 (37.9%)	0.214
Test to evaluate for MPN	22/115 (19.1%)	3/29 (10.3%)	0.410
Test to evaluate for MDS/MPN	8/115 (7.0%)	1/29 (3.45%)	0.687
Test to evaluate for BPDCN	1/115 (0.9%)	0/29 (0.00%)	1.000
Actionable results identified, yes/no (%)	75/115 (65.2%)	0/29 (0.00%)	<0.0001
Level 1 and/or 2 variants identified, yes/no (%)^a^	82/115 (71.3%)	14/29 (48.3%)	0.027
Level 1, yes/no (%)	69/115 (60.0%)	11/29 (37.9%)	0.038
Level 2, yes/no (%)	56/115 (48.7%)	6/29 (20.7%)	0.007
*FLT3* ITD or D835 variant among those with FLT3 variants identified on mutational analysis, yes/no (%)^b^	3/3 (100%)	0/0	
*JAK2* V617F variant among those with *JAK2* variants identified on mutational analysis, yes/no (%)^b^	5/6 (83%)	0/0	

Abbreviations: AML, acute myeloid leukemia; MDS, myelodysplasia; MPN, myeloproliferative neoplasm; MRD, minimal/measurable residual disease.

^a^ Identified as level 1 and/or 2 variants on the NGS panel; % positive refers to the percentage of tests identifying at least one variant in a gene of interest.

^b^ Results from separate *FLT3* and *JAK2* mutation assays; if performed more than once, the analysis performed most recently in relation to the NGS order was selected.

For patients with a history of or a new diagnosis of acute myeloid leukemia (AML) at the time of ordering (*n* = 52), the most commonly mutated genes were *RUNX1, SRSF2*, and *IDH2* (each with 16 variants). For patients with a history of or a new diagnosis of MDS at the time of ordering (*n* = 35), the most commonly mutated genes were *TP53*, *TET2*, and *SF3B1* with 11, 9, and 4 variants, respectively. Among tested patients with a history of or a newly established MPN (*n* = 9), the most commonly mutated genes were *JAK2* (six variants) and *MPL* (three variants). There were no triple‐negative MPNs; seven of nine patients (77.8%) demonstrated additional non‐driver variants; among the non‐driver variants reported to adversely affect overall survival in MPNs, [3, 14, 15] *SRSF2*, *ASXL1*, *EZH2*, and *SF3B1* variants were present (one of each). An independent *JAK2* V617F assay confirmed positivity in five of the six NGS‐demonstrated cases.

If the 29 Group B tests had been canceled up‐front, $24,600 could have been saved based on current Centers for Medicare and Medicaid Services rates [16]. In combination with the 30 tests in Group C, this equates to savings of approximately $150,370 annually and $751,800 every 5 years.

## 4 DISCUSSION AND CONCLUSION

The likelihood of NGS identifying actionable results for hematologic neoplasms is correlated with particular clinical indications [17], supporting the idea that clinical indications can be objectively used to determine whether to perform MN‐NGS testing. One recent study found that NGS was not cost‐effective when it is employed for the clinical indication of neutropenia and/or thrombocytopenia [17]. Using our proposed approval and cancelation criteria, we found that one third (59/174) of all MN‐NGS orders could be canceled without missing any actionable variant, thereby not compromising patient care. Among the NGS tests that were performed but met our cancelation criteria, no clinically actionable results were identified; hence, our criteria appropriately exclude unnecessary NGS testing. Considering that NGS testing and reporting is time‐ and resource‐intensive, implementing our screening criteria for MN‐NGS testing may result in more directed and cost‐efficient evaluation of MNs. Larger studies to confirm this finding are warranted; still, we determined that cost savings at a single institution were more than $150,000 annually and that number is likely to grow as NGS testing becomes more widely utilized.

Assessing for MRD in AML patients using NGS following induction has been shown to offer important prognostic information [7, 18], but the clinical utility of NGS for assessing MRD in the pre‐transplant setting, regardless of time following induction, has yet to be determined. Additionally, donor chimerism thresholds for MRD assessment by NGS in the post‐transplant setting need to be investigated. Once there is a wide‐spread implementation of error‐corrected sequencing as developed by Schmitt et al. [19], it may be prudent to consider expanding approval criteria to include pre‐ and/or post‐transplant MRD evaluation for AML as a valid indication for NGS; MRD identification by error‐corrected NGS has been shown to be highly predictive of AML relapse and overall survival due to its higher sensitivity, compared to conventional NGS [20]. However, some detected somatic variants can persist without affecting the relapse rate, for example, *DNMT3A* [21, 22]. Furthermore, predicting the risk of relapse will only be clinically useful if clinical management is altered as a result of testing [23].

One potential limitation of implementing our proposed criteria is that for post‐induction MRD assessment, the marrow NGS panel is not infrequently ordered concurrently with flow cytometry and surgical pathology assessment; hence, the presence or absence of immunophenotypic and/or morphologic evidence of measurable disease may not yet be known before the NGS order can be approved or denied. Cases such as these were still included in our Group A under criterion #6 so as to not exclude potentially important NGS orders. It may be reasonable (at the discretion of the ordering provider and pathologist) to perform NGS once flow cytometric and/or cytomorphologic analysis is complete. This same principle can be applied to the post‐transplant setting, where it may be reasonable to decide whether to perform NGS testing once chimerism studies are finalized. It should also be noted that in some cases, it can be challenging to definitively determine that there is no suspicion for an MN upfront (criterion #7); in these cases (depending on the clinical scenario), it may be prudent to extract the nucleic acids and hold the NGS sample until necessary ancillary studies have resulted.

Since this is a single‐institutional study with specific protocols and practices, these criteria may not be entirely applicable to other institutions. Larger, multi‐institutional studies are needed to refine criteria and create more generalizable recommendations.

## AUTHOR CONTRIBUTIONS

Savanah D. Gisriel conceptualized content, collected data, and drafted the manuscript. John G. Howe, Christopher A. Tormey, Richard Torres, Karl M. Hager, and Henry M. Rinder conceptualized content and edited the manuscript. Alexa J. Siddon conceptualized content, edited the manuscript, and provided project oversight.

## CONFLICT OF INTEREST STATEMENT

The authors declare no conflicts of interest.

## FINDING INFORMATION

The authors do not declare any funding sources.

## ETHICS STATEMENT

This study was conducted in compliance with institutional ethics policies.

## PATIENT CONSENT STATEMENT

The authors have confirmed patient consent statement is not needed for this submission.

## CLINICAL TRIAL REGISTRATION

The authors have confirmed clinical trial registration is not needed for this submission.

## Data Availability

For original deidentified data, please contact Savanah Gisriel at sgisriel@wisc.edu.
